# Supplementary material for: Exotic lagomorph may influence eagle abundances and breeding spatial aggregations: a field study and meta-analysis on the nearest neighbor distance
Source: PeerJ. 2018 May 10;6:e4746. doi: 10.7717/peerj.4746 (PMC5949207; doi:10.7717/peerj.4746)

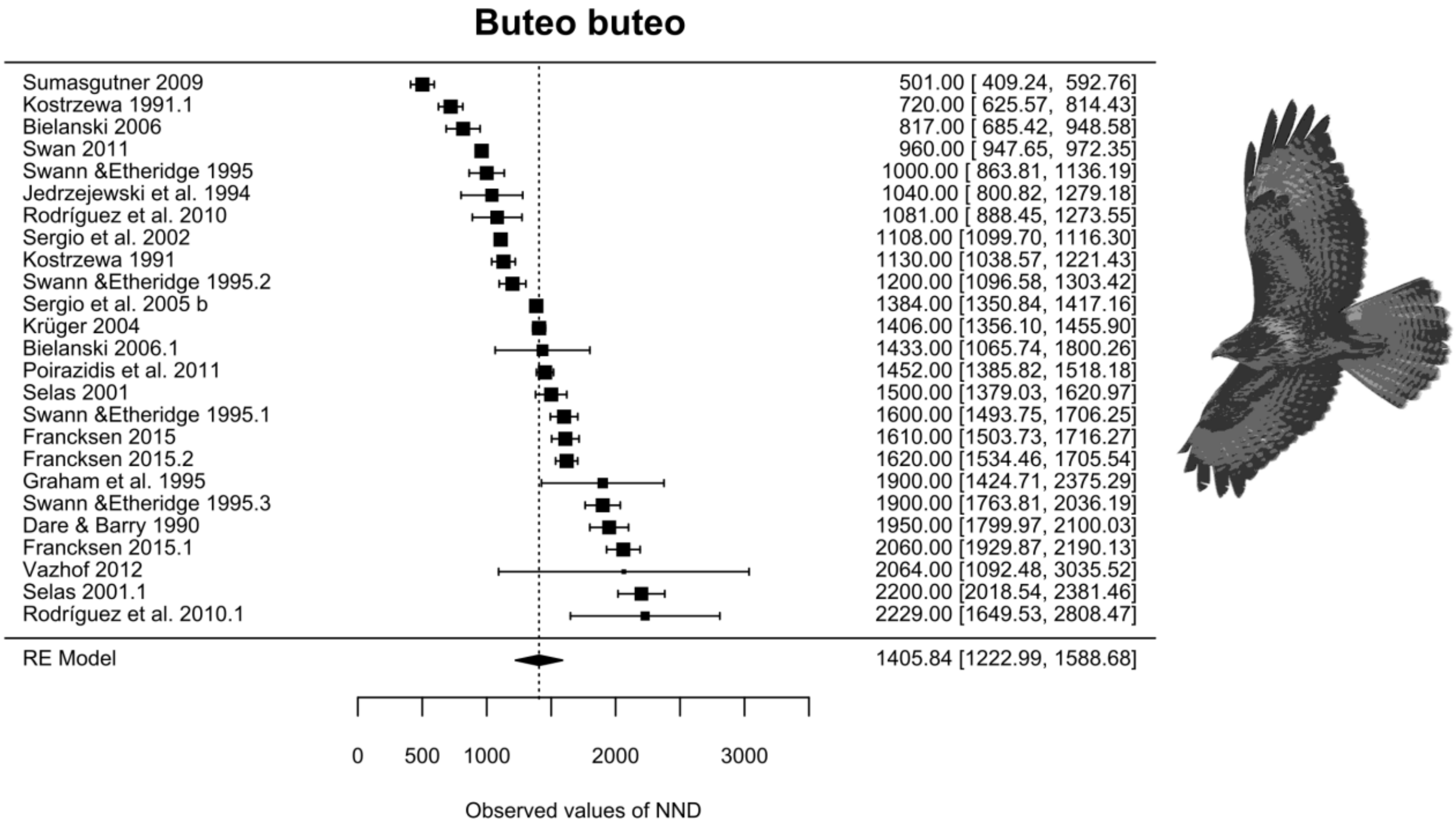
Buteo buteo
Sumasgutner 2009 501.00 [409.24, 592.76]
Kostrzewa 1991.1 720.00 [625.57, 814.43]
Bielanski 2006 817.00 [685.42, 948.58]
Swan 2011 960.00 [947.65, 972.35]
Swann &Etheridge 1995 1000.00 [863.81, 1136.19]
Jedrzejewski et al. 1994 1040.00 [800.82, 1279.18]
Rodríguez et al. 2010 1081.00 [888.45, 1273.55]
Sergio et al. 2002 1108.00 [1099.70, 1116.30]
Kostrzewa 1991 1130.00 [1038.57, 1221.43]
Swann &Etheridge 1995.2 1200.00 [1096.58, 1303.42]
Sergio et al. 2005 b 1384.00 [1350.84, 1417.16]
Krüger 2004 1406.00 [1356.10, 1455.90]
Bielanski 2006.1 1433.00 [1065.74, 1800.26]
Poirazidis et al. 2011 1452.00 [1385.82, 1518.18]
Selas 2001 1500.00 [1379.03, 1620.97]
Swann &Etheridge 1995.1 1600.00 [1493.75, 1706.25]
Francksen 2015 1610.00 [1503.73, 1716.27]
Francksen 2015.2 1620.00 [1534.46, 1705.54]
Graham et al. 1995 1900.00 [1424.71, 2375.29]
Swann &Etheridge 1995.3 1900.00 [1763.81, 2036.19]
Dare & Barry 1990 1950.00 [1799.97, 2100.03]
Francksen 2015.1 2060.00 [1929.87, 2190.13]
Vazhof 2012 2064.00 [1092.48, 3035.52]
Selas 2001.1 2200.00 [2018.54, 2381.46]
Rodríguez et al. 2010.1 2229.00 [1649.53, 2808.47]
RE Model 1405.84 [1222.99, 1588.68]
0
500
1000
2000
3000
Observed values of NND

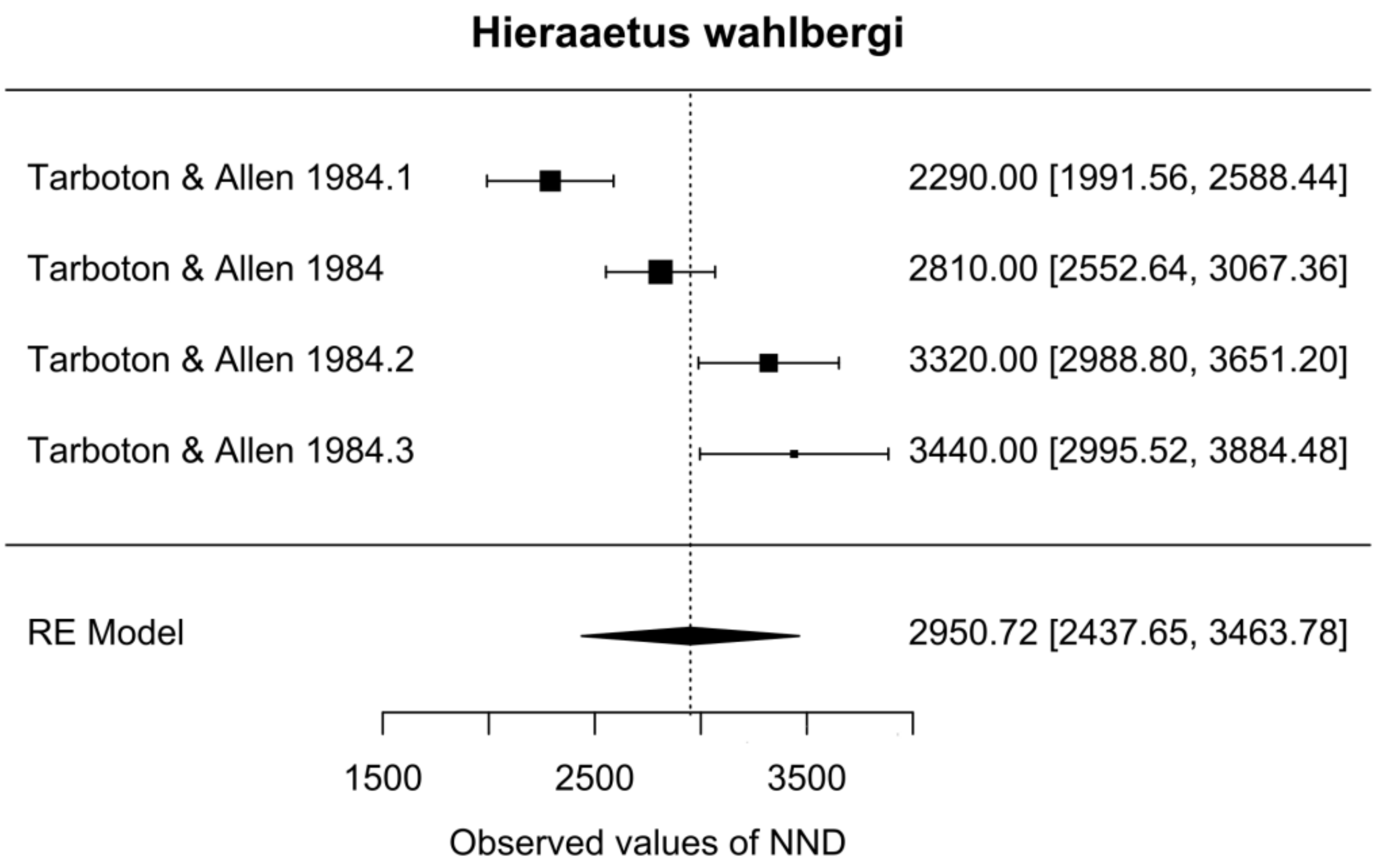
Hieraaetus wahlbergi
Tarboton & Allen 1984.1
2290.00 [1991.56, 2588.44]
Tarboton & Allen 1984
2810.00 [2552.64, 3067.36]
Tarboton & Allen 1984.2
3320.00 [2988.80, 3651.20]
Tarboton & Allen 1984.3
3440.00 [2995.52, 3884.48]
RE Model
2950.72 [2437.65, 3463.78]
1500
2500
3500
Observed values of NND

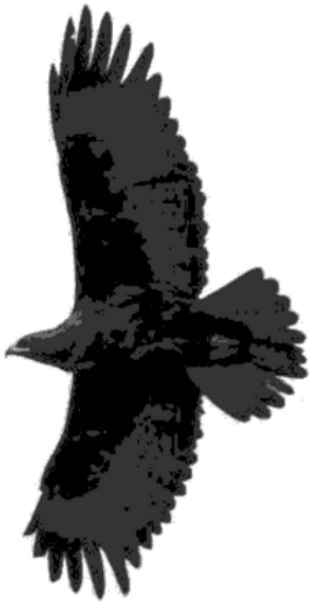

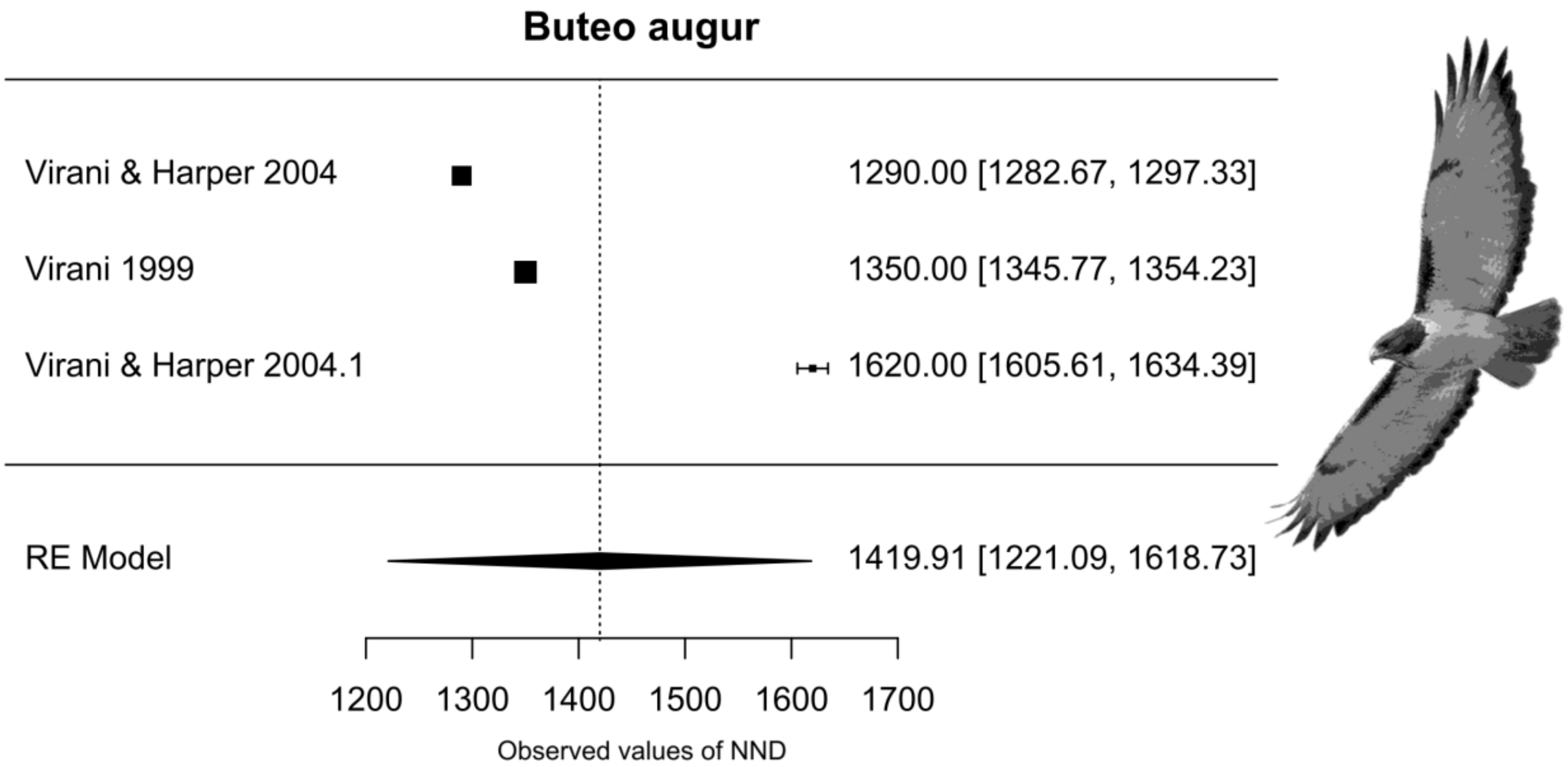

Buteo augur
Virani & Harper 2004
1290.00 [1282.67, 1297.33]
Virani 1999
1350.00 [1345.77, 1354.23]
Virani & Harper 2004.1
1620.00 [1605.61, 1634.39]
RE Model
1419.91 [1221.09, 1618.73]
1200
1300
1400
1500
1600
1700
Observed values of NND

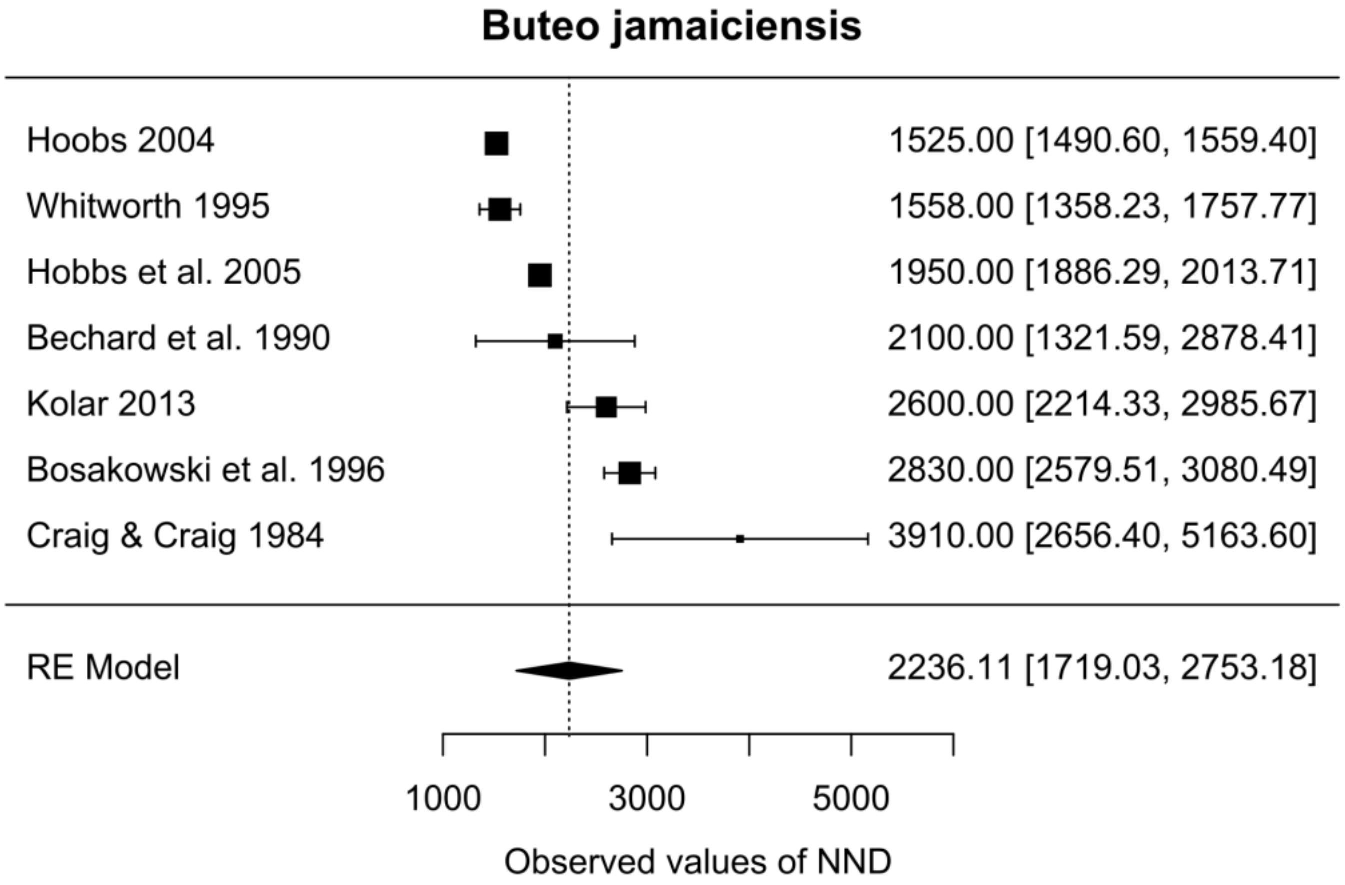
Buteo jamaiciensis
Hoobs 2004
Whitworth 1995
Hobbs et al. 2005
Bechard et al. 1990
Kolar 2013
Bosakowski et al. 1996
Craig & Craig 1984
RE Model
1525.00 [1490.60, 1559.40]
1558.00 [1358.23, 1757.77]
1950.00 [1886.29, 2013.71]
2100.00 [1321.59, 2878.41]
2600.00 [2214.33, 2985.67]
2830.00 [2579.51, 3080.49]
3910.00 [2656.40, 5163.60]
2236.11 [1719.03, 2753.18]
1000
3000
5000
Observed values of NND

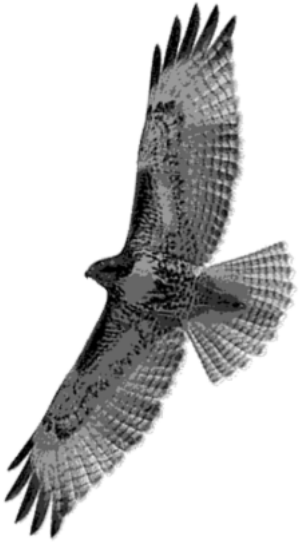

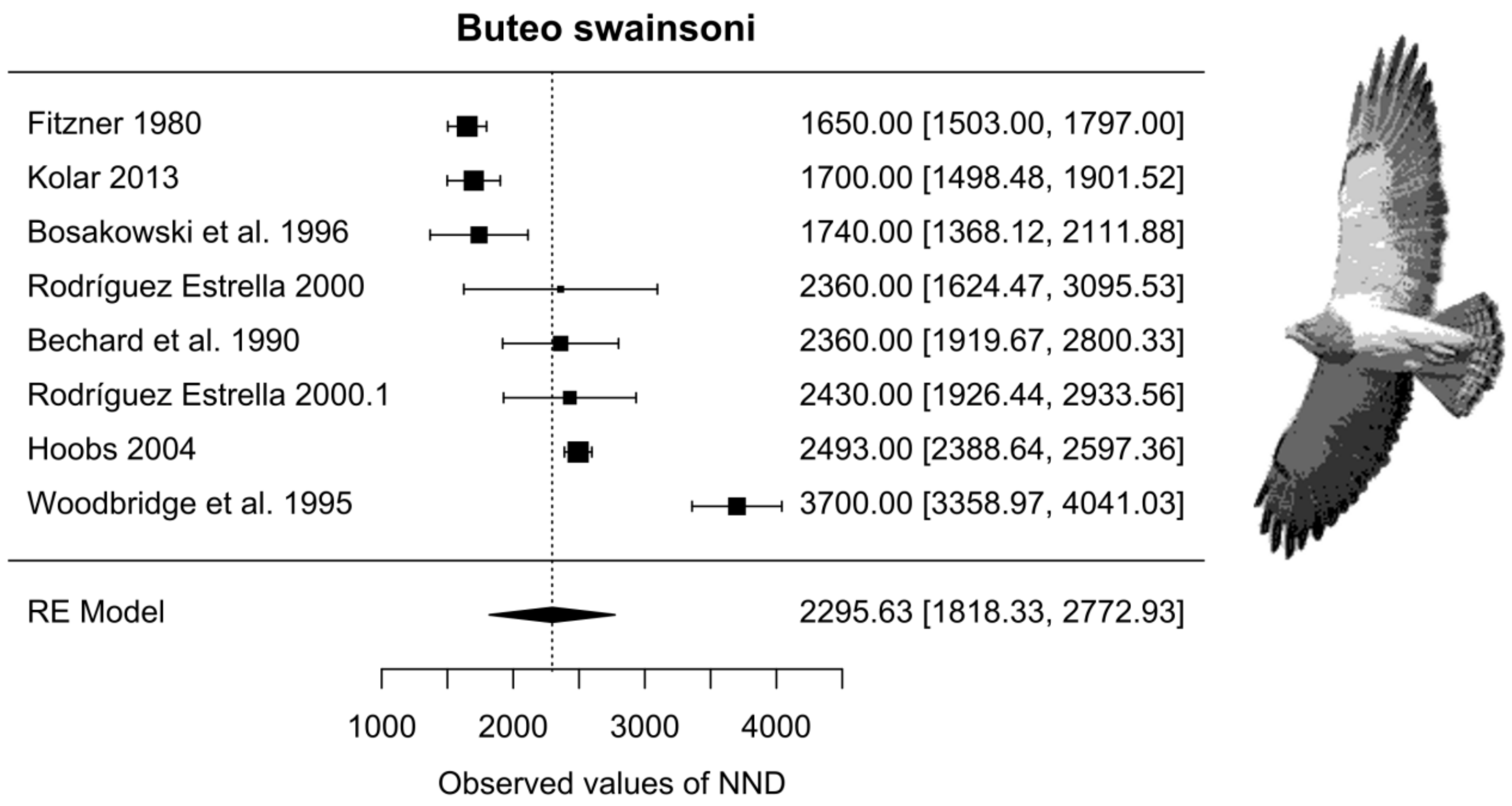

Buteo swainsoni
Fitzner 1980 1650.00 [1503.00, 1797.00]
Kolar 2013 1700.00 [1498.48, 1901.52]
Bosakowski et al. 1996 1740.00 [1368.12, 2111.88]
Rodríguez Estrella 2000 2360.00 [1624.47, 3095.53]
Bechard et al. 1990 2360.00 [1919.67, 2800.33]
Rodríguez Estrella 2000.1 2430.00 [1926.44, 2933.56]
Hoobs 2004 2493.00 [2388.64, 2597.36]
Woodbridge et al. 1995 3700.00 [3358.97, 4041.03]
RE Model 2295.63 [1818.33, 2772.93]
1000
2000
3000
4000
Observed values of NND

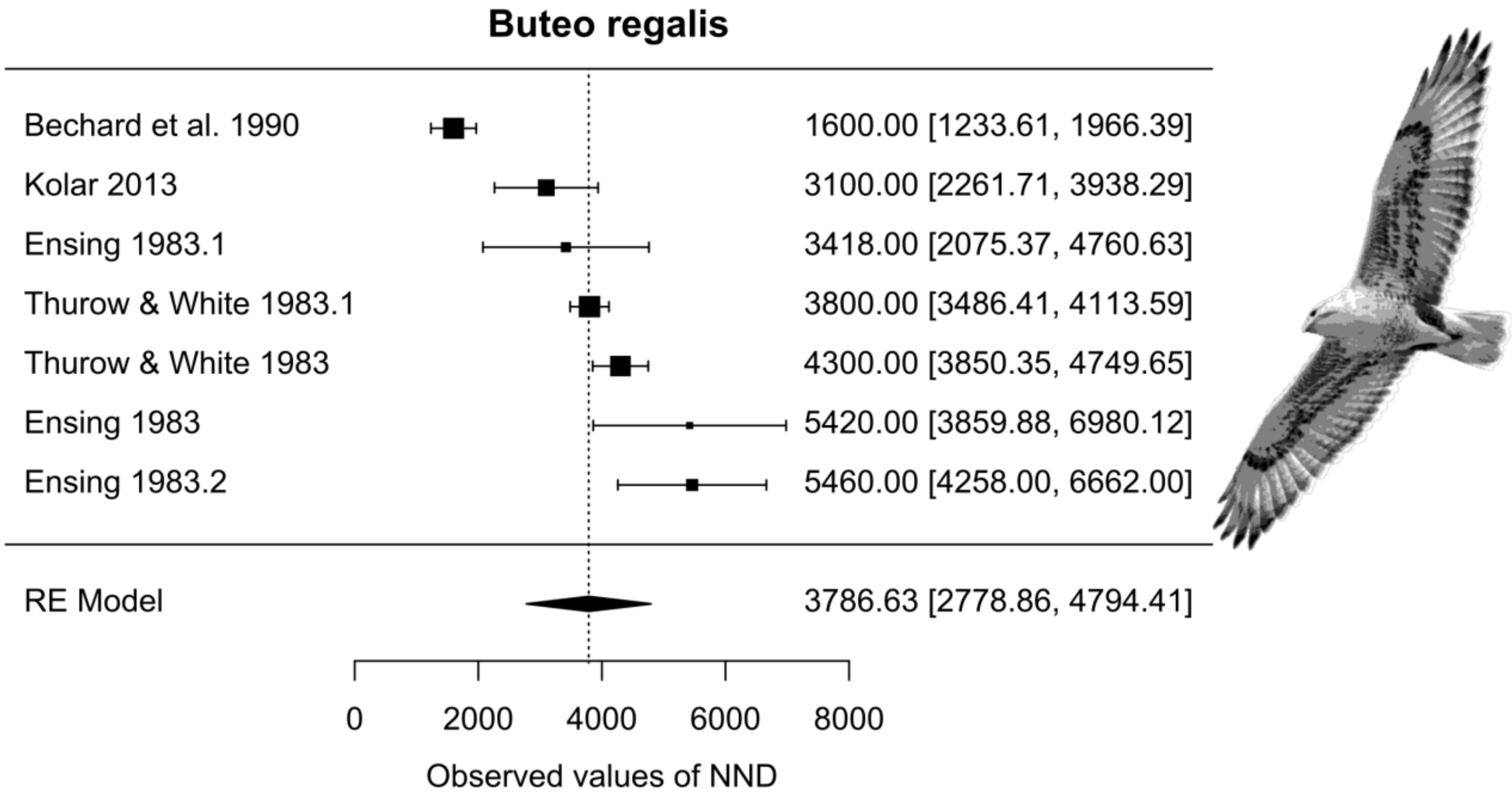
Buteo regalis
Bechard et al. 1990
Kolar 2013
Ensing 1983.1
Thurow & White 1983.1
Thurow & White 1983
Ensing 1983
Ensing 1983.2
RE Model
1600.00 [1233.61, 1966.39]
3100.00 [2261.71, 3938.29]
3418.00 [2075.37, 4760.63]
3800.00 [3486.41, 4113.59]
4300.00 [3850.35, 4749.65]
5420.00 [3859.88, 6980.12]
5460.00 [4258.00, 6662.00]
3786.63 [2778.86, 4794.41]
0
2000
4000
6000
8000
Observed values of NND

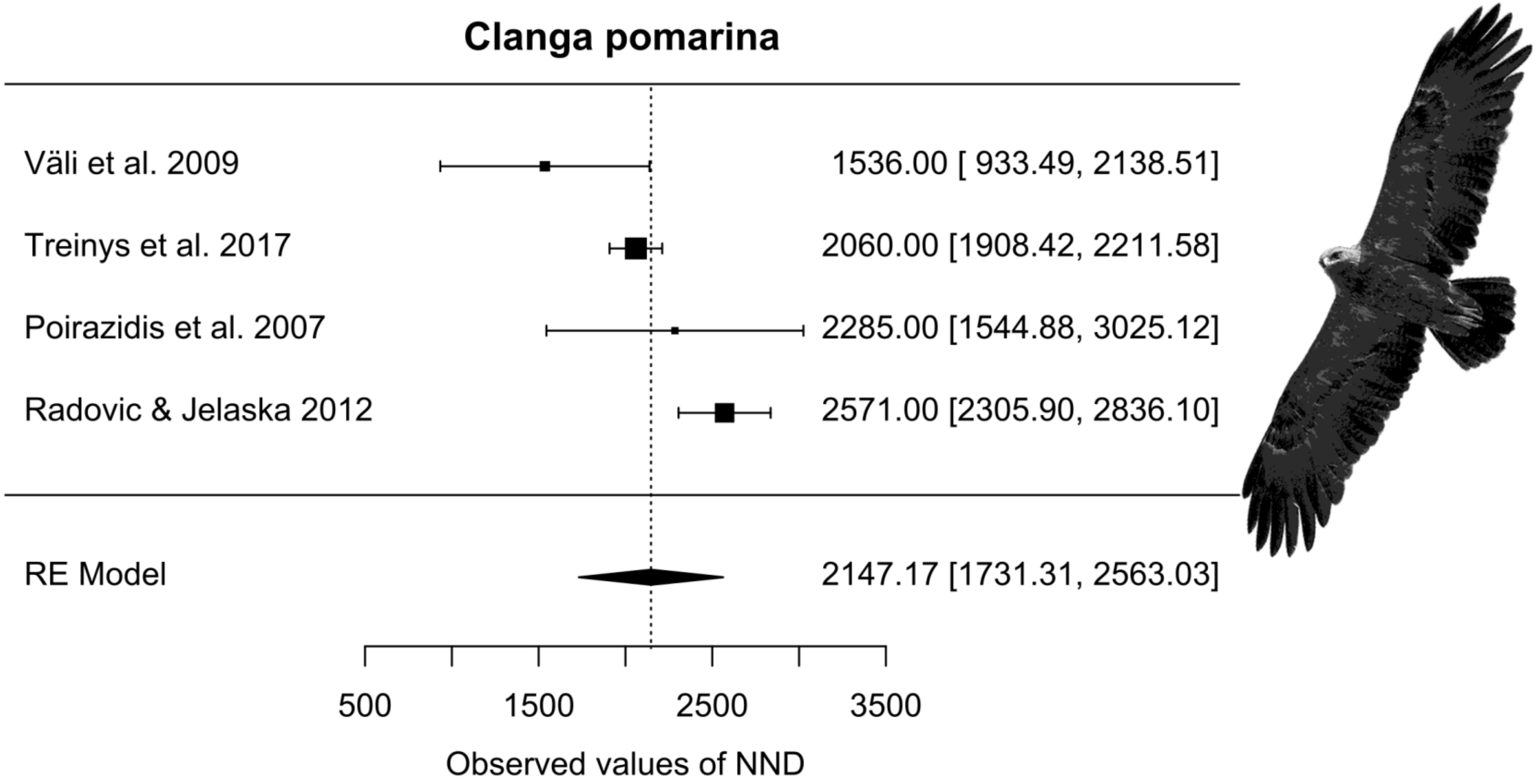

Clanga pomarina
Väli et al. 2009
1536.00 [933.49, 2138.51]
Treinys et al. 2017
2060.00 [1908.42, 2211.58]
Poirazidis et al. 2007
2285.00 [1544.88, 3025.12]
Radovic & Jelaska 2012
2571.00 [2305.90, 2836.10]
RE Model
2147.17 [1731.31, 2563.03]
500
1500
2500
3500
Observed values of NND

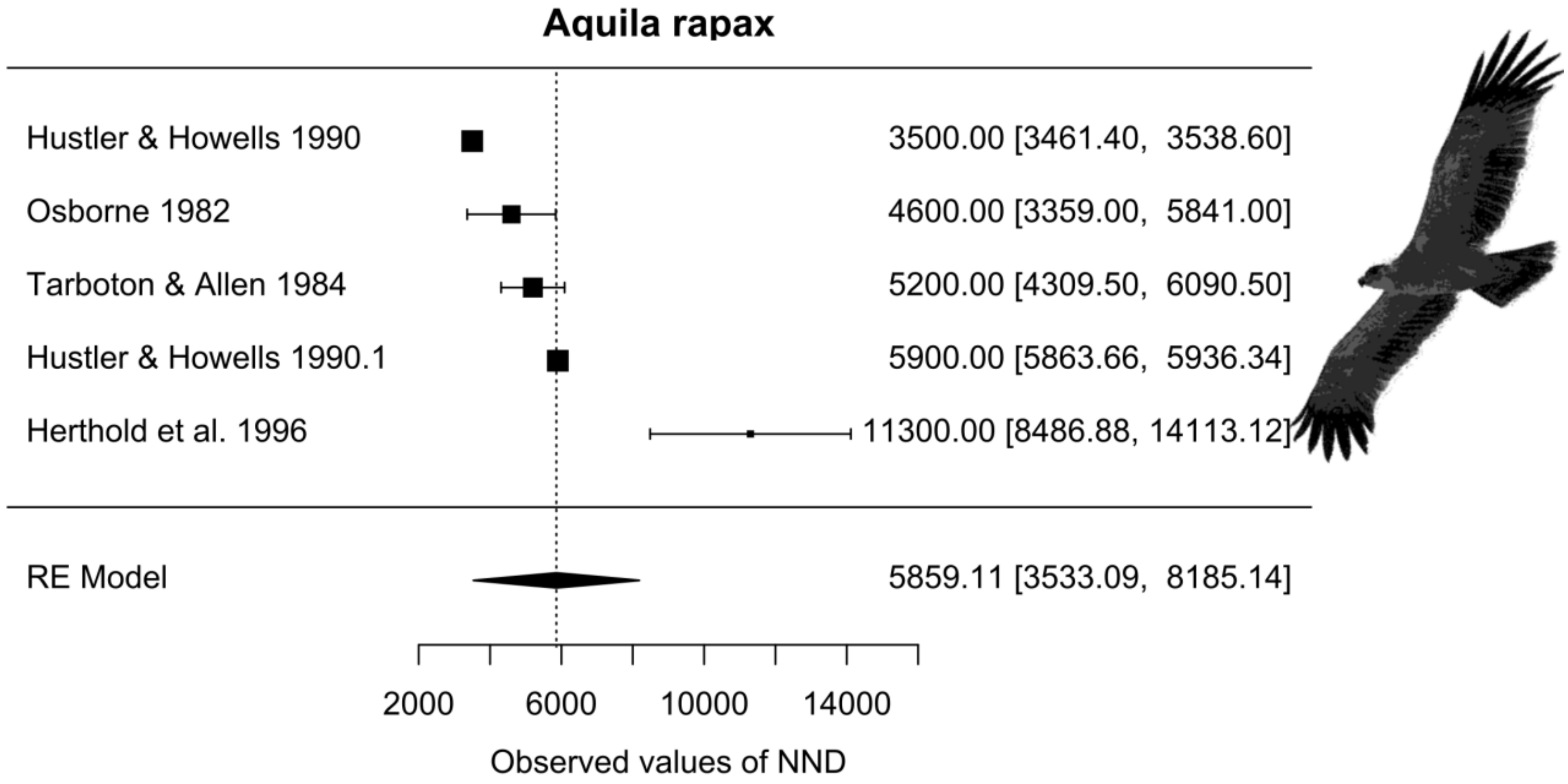

Aquila rapax
Hustler & Howells 1990
3500.00 [3461.40, 3538.60]
Osborne 1982
4600.00 [3359.00, 5841.00]
Tarboton & Allen 1984
5200.00 [4309.50, 6090.50]
Hustler & Howells 1990.1
5900.00 [5863.66, 5936.34]
Herthold et al. 1996
11300.00 [8486.88, 14113.12]
RE Model
5859.11 [3533.09, 8185.14]
2000
6000
10000
14000
Observed values of NND

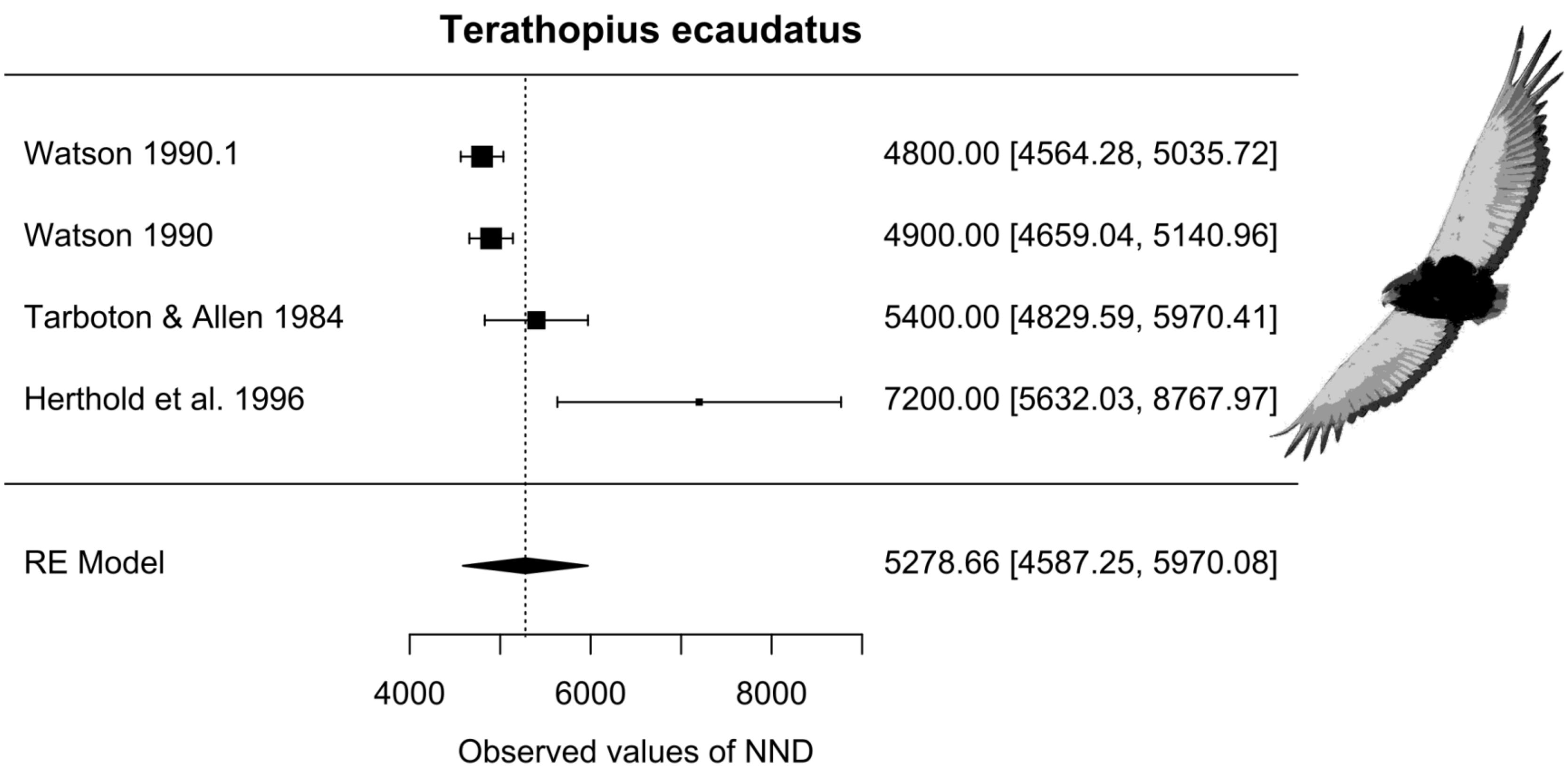
Terathopius ecaudatus
Watson 1990.1
4800.00 [4564.28, 5035.72]
Watson 1990
4900.00 [4659.04, 5140.96]
Tarboton & Allen 1984
5400.00 [4829.59, 5970.41]
Herthold et al. 1996
7200.00 [5632.03, 8767.97]
RE Model
5278.66 [4587.25, 5970.08]
4000
6000
8000
Observed values of NND

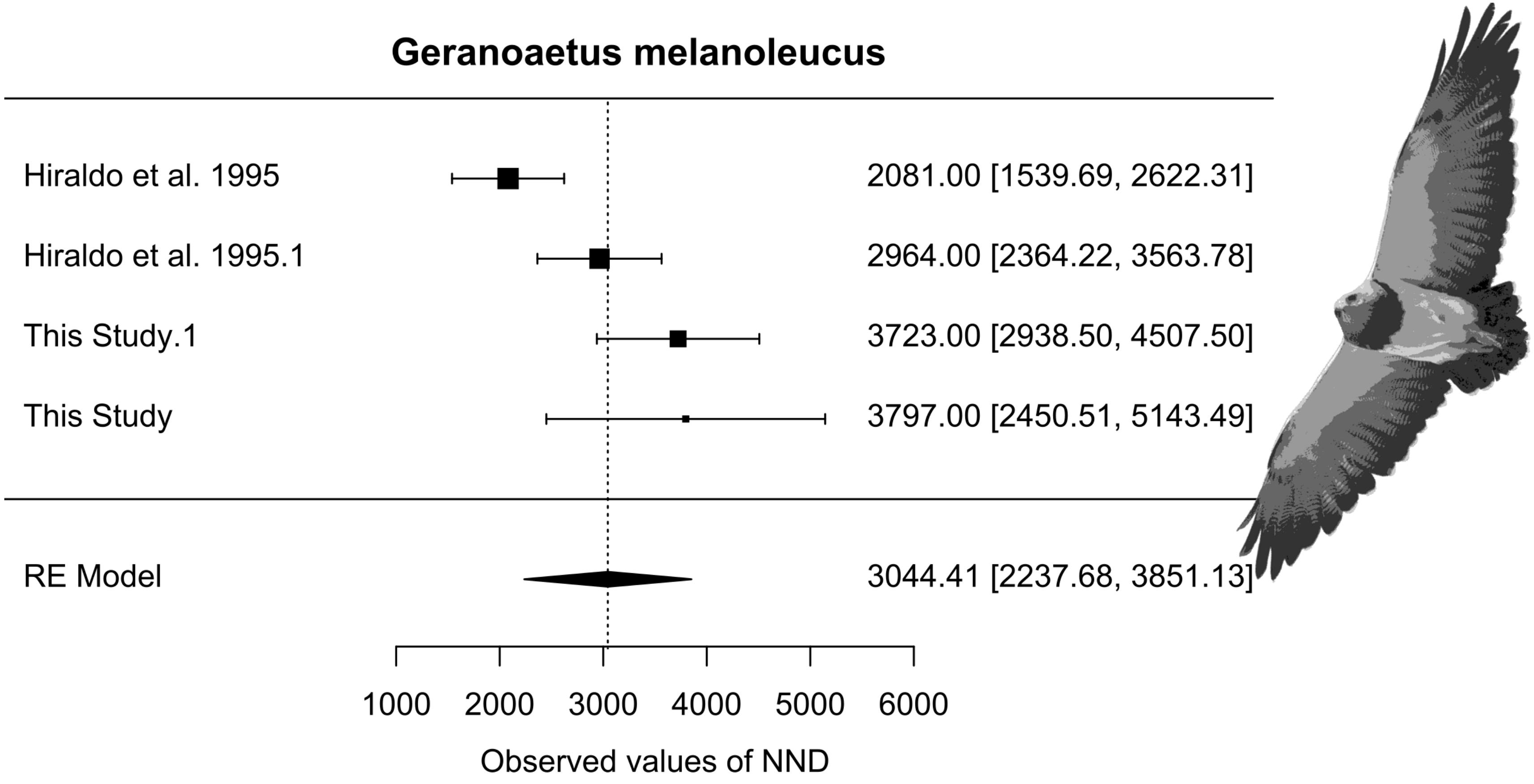
Geranoaetus melanoleucus
Hiraldo et al. 1995
2081.00 [1539.69, 2622.31]
Hiraldo et al. 1995.1
2964.00 [2364.22, 3563.78]
This Study.1
3723.00 [2938.50, 4507.50]
This Study
3797.00 [2450.51, 5143.49]
RE Model
3044.41 [2237.68, 3851.13]
1000
2000
3000
4000
5000
6000
Observed values of NND

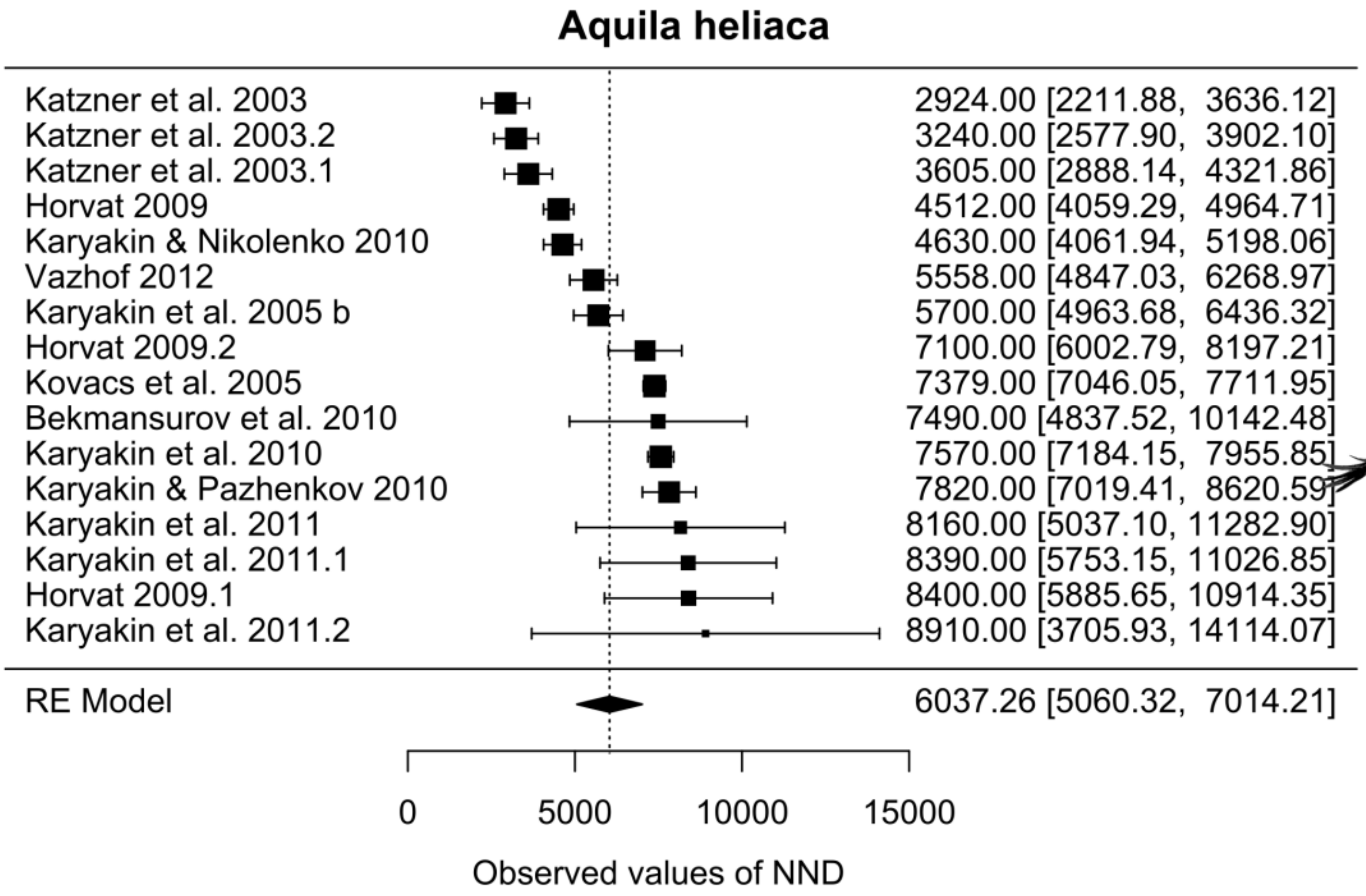
Aquila heliaca
Katzner et al. 2003
Katzner et al. 2003.2
Katzner et al. 2003.1
Horvat 2009
Karyakin & Nikolenko 2010
Vazhof 2012
Karyakin et al. 2005 b
Horvat 2009.2
Kovacs et al. 2005
Bekmansurov et al. 2010
Karyakin et al. 2010
Karyakin & Pazhenkov 2010
Karyakin et al. 2011
Karyakin et al. 2011.1
Horvat 2009.1
Karyakin et al. 2011.2
2924.00 [2211.88, 3636.12]
3240.00 [2577.90, 3902.10]
3605.00 [2888.14, 4321.86]
4512.00 [4059.29, 4964.71]
4630.00 [4061.94, 5198.06]
5558.00 [4847.03, 6268.97]
5700.00 [4963.68, 6436.32]
7100.00 [6002.79, 8197.21]
7379.00 [7046.05, 7711.95]
7490.00 [4837.52, 10142.48]
7570.00 [7184.15, 7955.85]
7820.00 [7019.41, 8620.59]
8160.00 [5037.10, 11282.90]
8390.00 [5753.15, 11026.85]
8400.00 [5885.65, 10914.35]
8910.00 [3705.93, 14114.07]
RE Model
6037.26 [5060.32, 7014.21]
0
5000
10000
15000
Observed values of NND

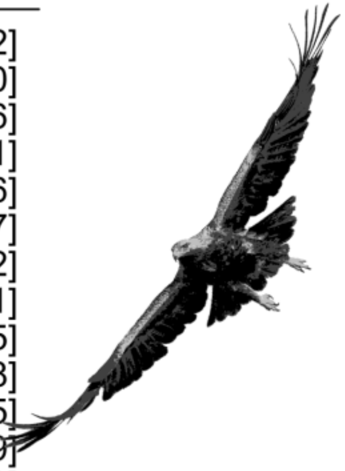

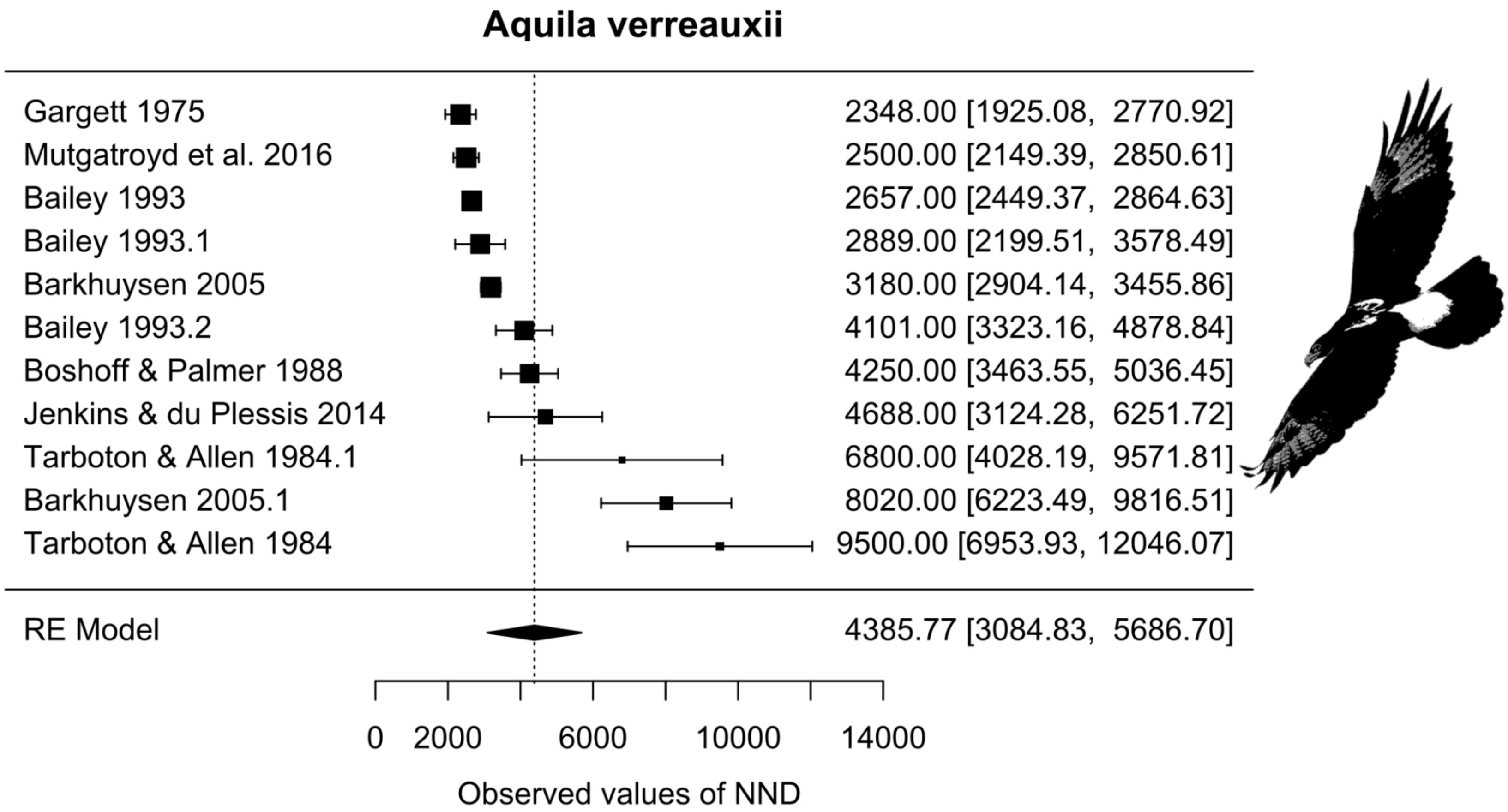
Aquila verreauxii
Gargett 1975 2348.00 [1925.08, 2770.92]
Mutgatroyd et al. 2016 2500.00 [2149.39, 2850.61]
Bailey 1993 2657.00 [2449.37, 2864.63]
Bailey 1993.1 2889.00 [2199.51, 3578.49]
Barkhuysen 2005 3180.00 [2904.14, 3455.86]
Bailey 1993.2 4101.00 [3323.16, 4878.84]
Boshoff & Palmer 1988 4250.00 [3463.55, 5036.45]
Jenkins & du Plessis 2014 4688.00 [3124.28, 6251.72]
Tarboton & Allen 1984.1 6800.00 [4028.19, 9571.81]
Barkhuysen 2005.1 8020.00 [6223.49, 9816.51]
Tarboton & Allen 1984 9500.00 [6953.93, 12046.07]
RE Model 4385.77 [3084.83, 5686.70]
0 2000 6000 10000 14000
Observed values of NND

Aquila chrysaetos

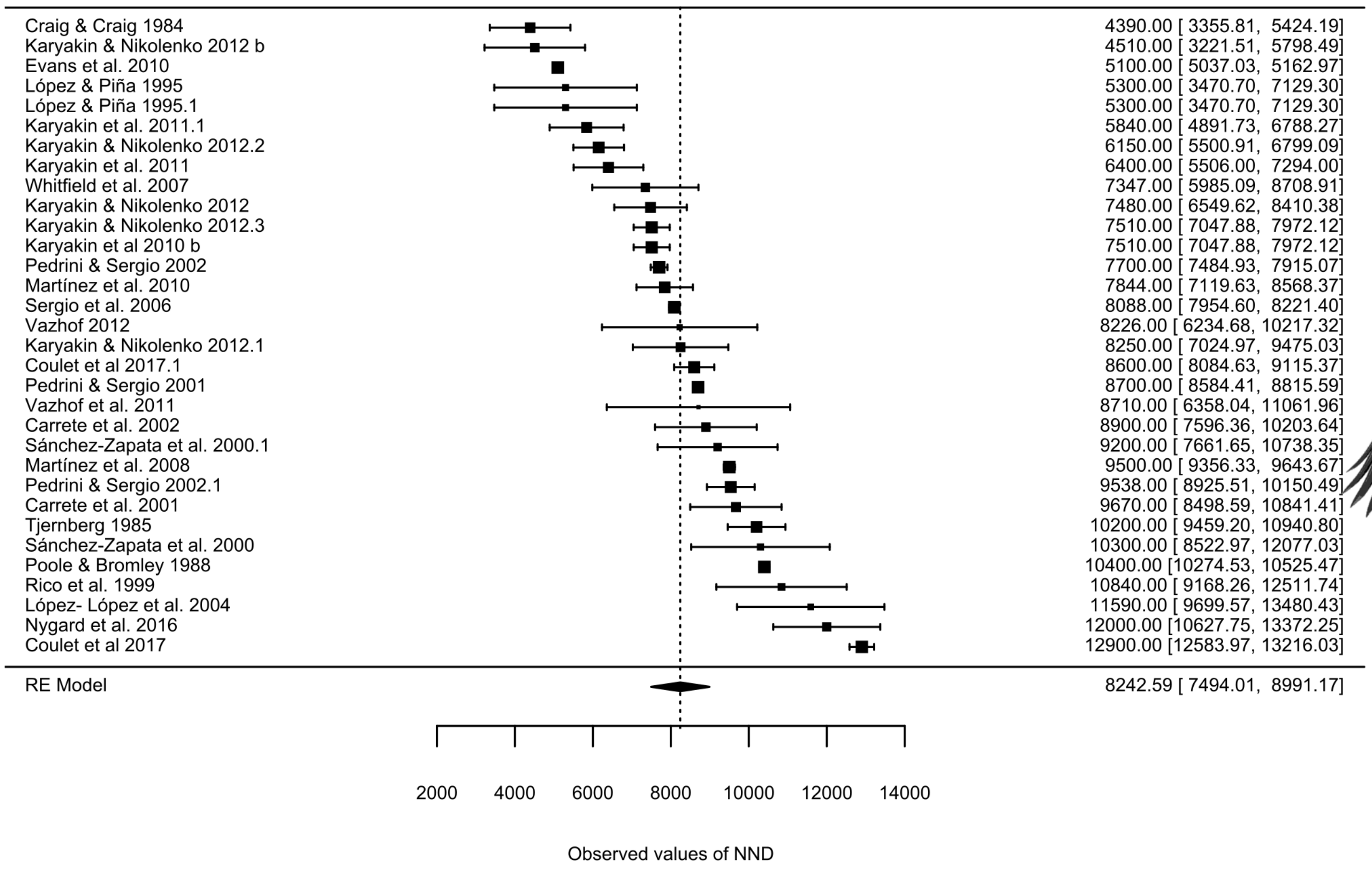

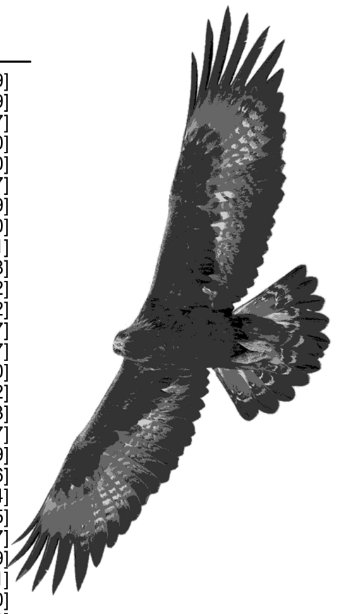

Supplement: Supplemental Information 4 — Every NND measure extracted from the papers found through our bibliogaphic search. The forest plots include the information of each study ID, NND and SD, later used for the calculation of NNDavg for each species. [file peerj-06-4746-s004.zip › Supporting file. Meta-analysis raw data IN FIGURES.pdf]
